# Interleukin-1 and acute brain injury

**DOI:** 10.3389/fncel.2015.00018

**Published:** 2015-02-06

**Authors:** Katie N. Murray, Adrian R. Parry-Jones, Stuart M. Allan

**Affiliations:** ^1^Faculty of Life Sciences, University of ManchesterManchester, UK; ^2^Salford Royal NHS Foundation TrustSalford, UK

**Keywords:** interleukin-1, inflammation, acute brain injury, cerebrovasculature

## Abstract

Inflammation is the key host-defense response to infection and injury, yet also a major contributor to a diverse range of diseases, both peripheral and central in origin. Brain injury as a result of stroke or trauma is a leading cause of death and disability worldwide, yet there are no effective treatments, resulting in enormous social and economic costs. Increasing evidence, both preclinical and clinical, highlights inflammation as an important factor in stroke, both in determining outcome and as a contributor to risk. A number of inflammatory mediators have been proposed as key targets for intervention to reduce the burden of stroke, several reaching clinical trial, but as yet yielding no success. Many factors could explain these failures, including the lack of robust preclinical evidence and poorly designed clinical trials, in addition to the complex nature of the clinical condition. Lack of consideration in preclinical studies of associated co-morbidities prevalent in the clinical stroke population is now seen as an important omission in previous work. These co-morbidities (atherosclerosis, hypertension, diabetes, infection) have a strong inflammatory component, supporting the need for greater understanding of how inflammation contributes to acute brain injury. Interleukin (IL)-1 is the prototypical pro-inflammatory cytokine, first identified many years ago as the endogenous pyrogen. Research over the last 20 years or so reveals that IL-1 is an important mediator of neuronal injury and blocking the actions of IL-1 is beneficial in a number of experimental models of brain damage. Mechanisms underlying the actions of IL-1 in brain injury remain unclear, though increasing evidence indicates the cerebrovasculature as a key target. Recent literature supporting this and other aspects of how IL-1 and systemic inflammation in general contribute to acute brain injury are discussed in this review.

## Introduction

Approximately 15 million people worldwide have a stroke every year, from which one third die and another third are permanently disabled (Corbyn, 2014). Ischemic stroke accounts for 80% of all strokes with the remaining 20% being composed of intracerebral hemorrhage (ICH) and subarachnoid hemorrhage (SAH). Traumatic brain injury (TBI) also falls under the category of acute central nervous system (CNS) injury and its pattern of injury evolves in a similar way to ischemic and hemorrhagic stroke. Currently there is only one drug option available to ischemic stroke patients, the thrombolytic agent recombinant tissue plasminogen activator (tPA), which disperses the clot in the occluded vessel. The primary limitation of tPA is that only 5–13% of the stroke population are eligible for treatment as intravenous (i.v) administration must occur within a 4.5 h time frame of stroke onset for the benefits of the drug to outweigh the risks of hemorrhage (Hacke and Lichy, 2008). Therefore, research over the last two decades has focused on neuroprotective strategies with approximately 1000 compounds being tested preclinically and almost 200 progressing to clinical trials (O’Collins et al., 2006; Minnerup et al., 2012), with no success. Despite these attempts to identify successful stroke treatments, the only pharmacological therapies currently in use are anti-platelet treatments for the general population (Chen et al., 2000) and thrombolysis for the select few (Wardlaw et al., 2012). Similarly, SAH and ICH treatment options are limited to a narrow therapeutic window (Xu et al., 2014; Zhou et al., 2014) thus necessitating an urgent need for new treatment options applicable to a wider spectrum of patients and at extended time points.

In response to these translational failures guidelines were introduced in an attempt to ensure that complete and comprehensive neuroprotection studies were performed before any agent made it to clinical trial i.e., The STAIR criteria (Stroke Therapy Academic Industry Roundtable (STAIR), 1999; Fisher et al., 2009; Albers et al., 2011). Alongside suggestions for improvements to experimental design and conduct, it was also recognized by the STAIR committee that advanced age and prevalent co-morbidities must also be considered and incorporated when modeling ischemic stroke as they increase stroke susceptibility and lead to poorer outcomes (Sieber et al., 2014; Wang et al., 2014). In particular, the contribution of co-morbidities to inflammation prior to and post-stroke is of key importance when determining outcomes after acute CNS injury.

## Inflammation and brain injury

Inflammation plays a key detrimental and reparative role in CNS injury and it is widely accepted that inflammatory events prior to and following an insult can have far reaching effects on susceptibility and patient outcome and recovery (VanGilder et al., 2014). Inflammation is an evolutionary-conserved defense strategy of the immune system that can be mounted in response to injury or infection. Acute inflammation is a rapid response to tissue injury and/or pathogens and is traditionally considered a beneficial mechanism to limit damage and evoke tissue repair and resolution of injury (Cuartero et al., 2013). Chronic inflammation conversely is generally associated with dysregulation of the immune system and often manifests itself as systemic inflammatory disease (Elkind, 2010). Ultimately, prolonged or unregulated inflammation, either chronic or acute, is detrimental to health and is particularly damaging if it occurs in close temporal proximity to a CNS insult. Following acute brain injury (e.g., stroke, TBI) a local and systemic inflammatory response is mounted which triggers inflammatory signaling cascades, increases in expression of transcriptional regulators and infiltration and activation of immune cells (Lian et al., 2012; Chu et al., 2014). This inflammatory response evolves over a number of days to amplify the ischemic lesion, but also to initiate tissue repair in the late post-ischemic phase (Iadecola and Anrather, 2011).

## The role of cytokines in stroke

As part of the inflammatory response to brain injury, chemokines and cytokines are secreted from immune cells to trigger a local pro- or anti-inflammatory response on surrounding target cells (Luheshi et al., 2009). Following a CNS insult, multiple cytokines are generated to cause, exacerbate, mediate and/or inhibit cellular injury and repair (Allan et al., 2005). The site of cytokine action is varied and cytokines can be expressed by or act upon glia, neurons, cerebrovascular endothelium and circulating immune cells (Allan and Rothwell, 2001). Under normal basal conditions cytokines are expressed at very low levels which are often difficult to quantify (Hopkins and Rothwell, 1995; Vitkovic et al., 2000). However, following CNS injury they are one of the first mediators relayed to the site of injury (Allan and Rothwell, 2003). The whole range of cytokine families (interleukins (IL), interferons (IFN), tumor necrosis factors (TNF), colony stimulating factors, growth factors and chemokines) have been implicated as contributors to pre-existing risk factors for stroke as well as following reperfusion (Fouda et al., 2013; Zhang et al., 2014). More specifically IL-1, IL-6, IL-10, IL-17, IL-23, TNFα, transforming growth factor β (TGFβ) and IFNγ are seen to increase after stroke (Lakhan et al., 2009), IL-17, IL-23 and IFNγ being associated with exacerbation of stroke in mice (Yilmaz et al., 2006; Shichita et al., 2009), whereas IL-10 and TGFβ are protective (Spera et al., 1998; Zhu et al., 2002). Release of these cytokines generates an inflammatory cascade, resulting in the synthesis of various downstream mediators, including prostaglandin (PG)-E_2_, IL-6, nitric oxide (NO), IL-10 and neurotrophins (Pinteaux et al., 2002). IL-1, as the first member of the IL family described, is considered the prototypical inflammatory cytokine. This, together with extensive literature reporting actions of IL-1 in cerebral ischemia, means that this review will focus predominantly on IL-1. Discussion of other inflammatory mediators in stroke can be found in several recent articles—see (Doll et al., 2014) and (Lambertsen et al., 2012).

## Interleukin-1 and acute brain damage

**Figure 1:**
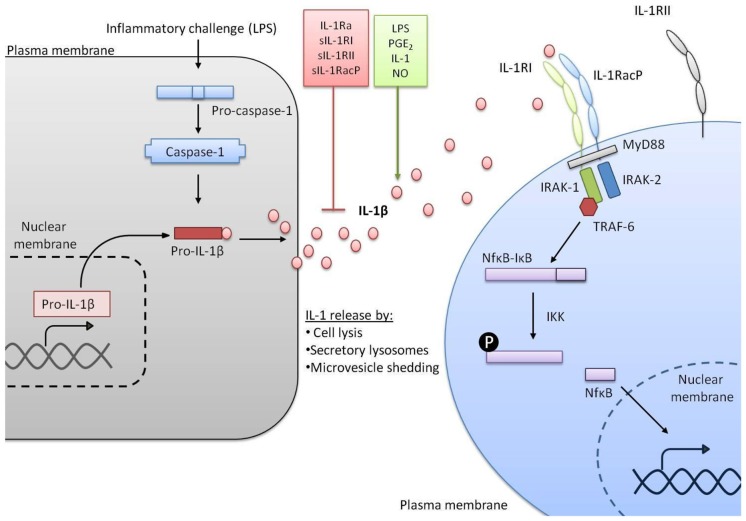
**IL-1 signaling pathways**. In response to a stimulus such as lipopolysaccharide (LPS) transcription of the gene encoding IL-1β is initiated. IL-1β is made as an inactive precursor protein and caspase-1 cleaves this pro-IL-1β to make the active IL-1β. A variety of factors can promote or inhibit the release of active IL-1β. Once released, IL-1β binds to IL-1RI alongside IL-1 receptor accessory protein (IL-1RAcP) and signal transduction is triggered, including co-localization of myeloid differentiation primary response protein 88 (MyD88), IL-1 receptor associated kinase (IRAK-1) and IRAK-2, recruitment of TNF receptor associated factor 6 (TRAF-6) and activation of nuclear factor kappa B (NFκB) from complex with IκB. Conversely IL-1 receptor type II (IL-1RII) does not induce signal transduction.

IL-1 is a key pro-inflammatory mediator with potent endogenous pyrogenic properties. IL-1 has been implicated in many pathological conditions, both in the periphery (e.g., sepsis, arthritis and autoimmune dysfunction), and centrally (e.g., TBI, SAH, ICH and cerebral ischemia). The two main IL-1 ligands are IL-1α and IL-1β, which show high sequence homology despite being products of different genes (Andrews et al., 1991; Figure 1). A third ligand, discovered in 1984, is a naturally occurring competitive antagonist, IL-1 receptor antagonist (IL-1Ra; Dinarello, 1994). This is highly selective and blocks all known actions of IL-1, with no known independent actions (Dinarello, 2011). IL-1 family members are constitutively expressed at low levels in the healthy brain and when released at modest concentrations locally, are not directly neurotoxic *in vitro* or *in vivo* (Lawrence et al., 1998; Rothwell and Luheshi, 2000), but play important roles in normal physiological processes such as development, sleep and synaptic plasticity as well as synaptic pruning and memory formation/consolidation during adulthood (del Rey et al., 2013).

Detrimental effects of IL-1 become evident when CNS injury occurs and there are raised levels of the cytokine. Acute neuronal injuries, such as stroke or TBI, cause a rapid up-regulation of IL-1β, IL-1Ra, IL-1 receptor (IL-1R) I, and IL-1RII expression in rats (Liu et al., 1993; Wang et al., 1997). Expression of IL-1α protein is also seen after cerebral ischemia, as early as 4 h post-reperfusion in microglial cells (Chen et al., 2007; Luheshi et al., 2011). Exogenous administration of recombinant IL-1β, either centrally or systemically, alongside experimental stroke in rodents leads to an exacerbation of ischemic damage (Yamasaki et al., 1995; Stroemer and Rothwell, 1998; McColl et al., 2007). Conversely, disruption of IL-1α and β activity in IL-1α/β knockout (KO) mice resulted in markedly reduced (70%) infarct volumes following experimental stroke (Boutin et al., 2001). Preclinical ICH and SAH studies also report increases in mRNA and protein expression of IL-1 following hemorrhagic injury (Wasserman et al., 2007; Greenhalgh et al., 2012), while clinical studies show that IL-1β promoter polymorphisms are associated with an increased risk of ICH in brain arteriovenous malformation patients (Kim et al., 2009). IL-1Ra has been shown to be safe in small Phase II trials in ischemic stroke (Emsley et al., 2005) and SAH, also resulting in a reduction in inflammatory markers in the circulation and cerebrospinal fluid (Singh et al., 2014). Ongoing clinical studies in larger patient cohorts will confirm the potential of IL-1Ra to move to Phase III efficacy trials.

## Pre-existing systemic inflammation and stroke incidence

Harmful effects of IL-1 are not limited to post-stroke inflammation. Accumulating evidence from the clinical and experimental setting suggests that pre-existing inflammation and elevated levels of IL-1 can also affect patient susceptibility and severity of CNS injury (McColl et al., 2009; Denes et al., 2010). The overwhelming majority of patients presenting with ischemic or hemorrhagic stroke have one or more risk factors including obesity, hypertension, atherosclerosis, diabetes and infection, which account for 60–80% of stroke risk in the general population (Hankey, 2006; Emsley and Hopkins, 2008). Alongside an increase in susceptibility to stroke, these risk factors also correlate to poorer outcomes both experimentally (Deng et al., 2014; Kim et al., 2014) and clinically (Oppenheimer et al., 1985; Razinia et al., 2007). Evidence indicates that a common element links all of these co-morbidities—namely a raised inflammatory status (Kwan et al., 2013). This pre-existing inflammation can present either chronically or as an acute event such as infection. The importance of these risk factors is highlighted by a study which showed that stroke incidence fell by 29% from 1999 to 2008 and 56 day mortality was reduced from 21% to 12% in 2008 due to better primary management of cardiovascular risk factors with lipid lowering and anti-hypertensive drugs (Lee et al., 2011). It is therefore essential to incorporate these conventional risk factors into preclinical models and to account for their potential actions when treating stroke patients.

Advancing age is the single most important non-modifiable risk factor for stroke with half of all ischemic events occurring in those aged over 75 (Roger et al., 2011). Tight control is usually exerted over the immune system; however, with advanced age this control is lost and there is an increase in serum levels of inflammatory cytokines (Jenny et al., 2002) which increases the vulnerability of the aged brain to stroke. In experimental models of stroke in aged, hypertensive and diabetic animals, an increase in mortality, neurological deficits and infarct volumes are observed (Rewell et al., 2010).

The metabolic syndrome which comprises obesity, dyslipidemia and diabetes is also a risk factor for stroke that has, with societal lifestyle changes, become increasingly prevalent in recent years (Mottillo et al., 2010). Obesity alone is an independent risk factor for stroke and a positive correlation has been observed in multiple ethnic populations and in both sexes, regardless of whether adiposity is measured by body mass index, waist circumference or waist to height proportion (Suk et al., 2003; Yatsuya et al., 2010; Bodenant et al., 2011). A raised systemic inflammatory profile is a characteristic feature of obesity, evidenced by the raised levels of c-reactive protein (CRP) and IL-6 (Visser et al., 1999; Yudkin et al., 1999). Furthermore, increasing circulating levels of IL-6 and CRP may lead to progressively higher risk of ischemic events (Rost et al., 2001; Miwa et al., 2013). Diabetic patients similarly have higher rates of mortality, more disabling strokes and exhibit impaired recovery following stroke in retrospective and prospective studies when compared to non-diabetic stroke patients (Pulsinelli et al., 1983; Oppenheimer et al., 1985; Woo et al., 1990).

Hypertension is another key modifiable stroke risk factor, with elevated blood pressure (BP) making up 30–40% of all ischemic stroke risk (Lawes et al., 2004). Presence of high BP prior to ischemia also resulted in worse outcome at 10 days and 6 months post-stroke when measured independently of baseline risk factors (Abboud et al., 2006). In experimental models, the harmful effects of obesity, diabetes and hypertension post-stroke have been clearly demonstrated, causing increased ischemic damage, greater disruption of blood brain barrier (BBB) integrity, increased occurrence of hemorrhagic transformation, greater neurological deficits and increased mortality (McColl et al., 2010; Li et al., 2013a,b). This exacerbation in acute injury due to the presence of a pre-existing inflammatory disease has also been seen in a model of ICH in the presence of experimental diabetes. Presence of hyperglycemia increased hematoma expansion and therefore resulted in worse outcome (Liu et al., 2011).

Atherosclerosis is one of the primary contributors to stroke risk due to the rupture and detachment of vascular plaques which can result in thromboembolism (Ohira et al., 2006). Inflammation plays a central role in the initiation and destabilization of atherosclerotic plaques. Unstable plaques have been shown to contain elevated levels of infiltrating leukocytes that express proteolytic enzymes and thrombogenic substances that contribute to the disruption of previously stable plaques (Ross, 1999; Patel et al., 2008; Packard et al., 2009). Experimental studies have utilized anti-inflammatory strategies (e.g., IL-1 neutralization, TNFα antagonism) to show that dampening of the inflammatory response hinders atherosclerotic lesion progression (Braunersreuther et al., 2008; McKellar et al., 2009; Bhaskar et al., 2011).

Infection is another critical risk factor for stroke, with epidemiological studies highlighting an association between bacterial or viral infection and ischemic stroke (Grau et al., 2010). In a study of approximately 19,000 patients from the UK general practice research database, the risk of first time stroke was highest 3 days after diagnosis of infection (Smeeth et al., 2004; Clayton et al., 2008). Further support for a link between infection and stroke is provided by research showing increased deaths attributable to cardiovascular disorders and stroke during respiratory infection epidemics (Eickhoff et al., 1961). Urinary and respiratory tract infections are most commonly associated with increased stroke risk, with *Streptococcus pneumoniae* and influenza both having firm associations (Grau et al., 1995). An increased incidence of hemorrhagic stroke has also been noted following upper respiratory infection due to the increased likelihood of formation and rupture of cerebral aneurisms, leading to SAH (Kunze et al., 2000). Furthermore, in a study examining incidence of infection in ICH patients, those that had infection had significantly larger hemorrhages, poorer National Institutes of Health Stroke Scale scores, raised levels of CRP and were more likely to experience intraventricular hemorrhage extension (Diedler et al., 2009). A causal relationship between stroke and infection is supported by overlap of pathways that are common to both, including inflammation and thrombosis. Platelet activation and aggregation is increased in venous blood samples from patients presenting with stroke and pre-existing infection vs. their non-infectious counterparts thus hinting at a potential common detrimental mechanisms (Zeller et al., 2005). Preclinical data on the relationship between stroke risk and infection are surprisingly sparse with only a small number of studies exploring the effects of infection on stroke outcome. One study showed that human influenza A infected mice had larger infarcts and greater disruption in BBB integrity in comparison to non-infected mice (Muhammad et al., 2011). Additionally, research within our own group has shown that chronic infection with the parasitic *Trichuris muris* model of gut infection prior to ischemic stroke in mice exhibited either a Th1 or Th2 polarized immune response. Mice with a Th1 response showed greater neurological deficits and exacerbation of ischemic brain injury (Dénes et al., 2010).

Common stroke risk factors often co-exist as they can converge on shared pathways (e.g., the inflammatory cascade) and therefore patients who have more than one of these risk factors are at a much greater risk of a severe ischemic event, as pre-existing co-morbidities may act synergistically to exacerbate damage (Howells et al., 2010). Since many of the systemic inflammatory conditions mentioned as risk factors for stroke can be improved by inhibition of IL-1, this suggests a key role for this pro-inflammatory cytokine in altering stroke susceptibility and severity.

## IL-1 and pre-existing inflammation

Clinically, elevated IL-1 levels are fundamental to many auto-inflammatory diseases which, as a result, may be improved by IL-1 neutralization (e.g., gout, osteoarthritis and post-myocardial infarction heart failure) (Dinarello, 2011). Growing evidence however, implicates this cytokine in known vascular risk factors for stroke (i.e., atherosclerosis, obesity, diabetes, infection and hypertension), and suggest it is crucial to disease progression in many experimental models of vascular disease (Murray et al., 2013).

### IL-1: a vascular risk factor

In atherosclerosis and obesity, NOD-like receptor protein (NLRP)-3 inflammasome (the inflammasome that controls caspase-1 activity and thus IL-1β processing to its mature form) was a key driving factor in progression of the diseases (Duewell et al., 2010; Vandanmagsar et al., 2011). These results are supported by research in a strain of atherosclerotic-susceptible mice (fed a high-fat diet) crossed with IL-1R1 KO mice. These mice, despite being predisposed to develop atherosclerosis, had a reduced plaque burden and lowered BP due to the ablation of the IL-1R1 and selective loss of IL-1 signaling (Chamberlain et al., 2009). A further study examining the inflammatory state of hypertensive rats following a stroke observed elevated levels of IL-1 which correlated to increased ischemic damage (Liu et al., 1993). In genetic association studies, IL-1 or IL-1Ra gene polymorphisms are associated with increased susceptibility to stroke, atherosclerosis and ICH in humans (Seripa et al., 2003; Um et al., 2003; Worrall et al., 2003; Dziedzic et al., 2005; Rezaii et al., 2009). Further genetic studies assessing the influence of IL-1 genotype status on the risk of cardiovascular disease show that patients with a predisposition to express higher levels of IL-1 were at a significantly higher risk of having coronary artery disease (CAD) due to excess oxidized phospholipids and lipoproteins. This enhanced risk of CAD was not observed in IL-1(-) genotypes (Tsimikas et al., 2014).

### IL-1 and exaggerated brain injury

In addition to increasing susceptibility to ischemic stroke, high levels of pre-existing IL-1, exacerbates post-stroke damage. Peripherally-administered lipopolysaccharide (LPS; which produces a robust IL-1 response) has been used to induce systemic inflammation in mice and administration prior to experimental middle cerebral artery occlusion results in a 150% increase in infarct volume when compared to vehicle-treated animals. To further confirm the importance of IL-1 in this model, animals treated with LPS and IL-1Ra had infarct volumes reduced by 60% compared to animals treated with LPS alone (McColl et al., 2007). Pre-existing IL-1 administration also exacerbates acute TBI injury by increasing volume of contusion injury, hippocampal neuronal death and enhancing perivascular neutrophil accumulation (Utagawa et al., 2008). Another example of the damaging effects of acute systemic IL-1 prior to ischemia is seen in models of infection. In mice and rats infected with *Streptococcus pneumoniae*, a robust IL-1 response was induced leading to larger infarct volumes, increased BBB disruption and functional deficits post-stroke. These effects were abrogated by delayed IL-1Ra administration (Dénes et al., 2014). Alongside preclinical evidence, clinical evidence also seems to hint that the presence of a pre-existing, inflammatory infection prior to stroke can impair outcome at later time points, as evidenced by neurological scores (Paganini-Hill et al., 2003; Grau et al., 2010).

The research outlined above indicates that systemic IL-1, whether it pre-existing or post-injury, plays a crucial role in mediating excess acute brain injury, though mechanisms involved remain unclear. As such we propose below a number of mechanisms through which IL-1 may mediate its detrimental actions in acute brain injury.

## Inflammation and the cerebrovasculature

During the acute phase of ischemic stroke, inflammation initiates a robust response from many cell types including glial and brain endothelial cells. Considering the vascular nature of stroke and that many of the risk factors that predispose patients to an ischemic insult are characterized by vascular inflammation, it is possible that the brain endothelium is a point of convergence for mechanisms of inflammatory-associated damage. The cerebrovasculature has a number of crucial roles in both physiological and pathological conditions, including regulation of vascular tone (Palomares and Cipolla, 2014). In situations where routes of flow are occluded or cerebral blood flow (CBF) is inadequate as in the case of ischemic and hemorrhagic stroke, intrinsic safeguards, both structural and functional in nature, are in place to maintain and stabilize CBF (Palomares and Cipolla, 2014).

### Structural abnormalities associated with vascular inflammation

Under pathological conditions risk factors for stroke have profound effects on cerebrovasculature structure with structural anomalies often being associated with chronic systemic inflammatory diseases (Arsava et al., 2014). In atherosclerosis, plaque formation reduces the internal diameter of vessels and increases the likelihood of thrombus formation and ischemic attack (Bogiatzi et al., 2014). In patients with hypertension, vascular remodeling and hypertrophy is a characteristic feature of the disease and contributes to increased wall thickness, reduced lumen diameter and reduced vascular responsiveness to stimuli (Pabbidi et al., 2013). Furthermore, in a small retrospective study of patients with chronic hyperglycemia, cerebral microvascular remodeling and perfusion deficits were observed in these patients through perfusion computer tomography (Hou et al., 2013). Further studies have also observed vascular asymmetry and a reduction in the number of branches in obese Zucker rats vs. lean Zucker and Wistar rats (Lapi et al., 2013).

### Functional abnormalities associated with vascular inflammation

In addition to changes in the structural architecture of the cerebrovasculature in the presence of systemic inflammation, functional deficits are also apparent. Experimentally, mice fed a high-fat diet for 8 weeks had impaired cerebrovascular function and neurovascular coupling leading to an increase in infarct volume and neurological deficits (Li et al., 2013c). In diabetic rats, CBF responses to sciatic nerve or whisker stimulation were depressed in both type I and type II diabetes (Jackman and Iadecola, 2015) The influence of inflammatory co-morbidities on ischemic penumbra has also been measured in stroke-prone spontaneously hypertensive rats (SHRSP) vs. Wistar Kyoto rats. Results from magnetic resonance imaging (MRI) showed that within 1 h of stroke, SHRSP had significantly more ischemic damage and a smaller penumbra than their normotensive counterparts (McCabe et al., 2009). The expanding perfusion deficit in SHRSP predicts more tissue at risk of infarction, which correlates to poorer clinical outcome. These results have important implications for management of stroke patients with pre-existing hypertension and suggest that ischemic damage could progress at a faster rate in the presence of a disease with an activated inflammatory cascade. It is likely that the vascular risk factors commonly associated with stroke cause cerebral vascular dysfunction (either structural and/or functional), which manifests as inadequate perfusion in brain areas at risk of infarction (the ischemic penumbra). In the clinical setting, perfusion deficits have also been observed in Alzheimer’s (Tosun et al., 2009; Austin et al., 2011) and Parkinson’s (Takahashi et al., 2010) disease patients and there is a positive correlation between disease progression and larger CBF deficits. Furthermore, cerebral hypoperfusion has been seen in both relapsing-remitting multiple sclerosis (MS) and primary MS (Adhya et al., 2006). Upregulation of vasoactive mediators have also been implicated in postmortem MS brain tissue and hypoperfusion has been observed in MS patients as measured by MRI (D’Haeseleer et al., 2013). Inflammation may therefore contribute to hypoperfusion in both acute and chronic pathologies in preclinical and clinical scenarios.

## Effects of acute IL-1 on cerebral blood flow

The cerebrovascular endothelium is highly responsive to pro-inflammatory stimuli and a primary location of IL-1RI, so it is possible that IL-1 could mediate changes in CBF observed in pathological disease states. Studies in rats have shown that prolonged intracerebroventricular (i.c.v) administration of IL-1 significantly reduced CBF (Maher et al., 2003). During early reperfusion in a rodent model of ischemic stroke a marked reduction in CBF was also observed in animals receiving an intraperitoneal (i.p) injection of IL-1. This same effect was not seen in control stroke animals who did not receive IL-1 (Parry-Jones et al., 2008). Whether effects on CBF reported with i.c.v injection of IL-1 (Maher et al., 2003) are as a result of systemic inflammatory changes is not known, since this was not assessed in the study. However, leakage of substances injected into the cerebral ventricles to the systemic circulation is known so it may be a possibility, especially given that IL-1 was administered over a 2- or 4-week period. A reduction in CBF in the cerebral microcirculation can impinge upon successful reperfusion thus leading to an accelerated collapse of the penumbra and expansion of infarct core. It is therefore possible, that the detrimental role of IL-1 on CBF in the early stages following acute stroke may account for the ability of IL-1 to exacerbate cerebral ischemia (Parry-Jones et al., 2008). In further studies examining this mechanism of IL-1 induced hypoperfusion, acute administration of IL-1 prior to ischemia resulted in a significant perfusion deficit and larger infarct volumes as measured by diffusion-weighted and perfusion-weighted MRI. It was revealed that raised levels of the vasoconstrictor endothelin-1 were present within tissue experiencing hypoperfusion and blockade of the endothelin receptor type A (ETrA) restored CBF and improved infarct volume and functional outcomes. Overall, this indicated acute systemic inflammation interacted with the vasculature to induce changes in CBF which ultimately had a detrimental effect during acute reperfusion (Murray et al., 2014). This hypothesis is further supported by translational studies demonstrating that patients with a history of recent acute infection in the week leading up to their stroke exhibited vascular dysfunction (Pleiner et al., 2004; Bryant et al., 2005). From a peripheral vascular perspective, infection can also transiently impair endothelium-dependent relaxation as observed in children with acute infections (generally upper respiratory tract). Brachial artery flow mediated dilation was measured in a cohort of 600 children suffering from acute infection or recovering from acute infection. Lower brachial artery flow was seen compared to uninfected controls (Charakida et al., 2005). Whilst not examined in the aforementioned human association studies, links between upper respiratory tract infection and high levels of IL-1 have been previously observed. In a study by Dénes et al. the presence of *Streptococcus pneumoniae* infection in mice and rats prior to ischemia significantly exacerbated infarct volume. Delayed administration of IL-1Ra however abolished the infection-induced deficits in functional outcomes and brain injury and vascular activation thus highlighting the detrimental effects of IL-1 on the cerebrovasculature prior to ischemia (Dénes et al., 2014).

## Chronic IL-1 and cerebral blood flow

Similar mechanisms of inflammation induced vasoconstriction have also been noted in a chronic inflammatory model, the obese Zucker rat in which pressure-induced vasoconstriction was examined. It was observed that these animals exhibited increased myogenic activation and a robust vasoconstrictive response vs. their lean counterparts. This phenomenon was abolished by removal of the endothelium, thus suggesting the endothelium was targeted by systemic inflammation and regulated arterial constriction (Butcher et al., 2013). Furthermore, depletion of macrophages in a hypertensive model improved perfusion however, peripheral arteries did not respond in a similar fashion, suggesting chronic inflammation has brain-specific effects which may not be mirrored in other vascular beds (Pires et al., 2013). Studies have shown that endothelium-dependent relaxation was impaired in type II diabetes in rats and could be restored using ETrA antagonism, thus reinforcing the concept that inflammation-induced vasoconstriction following ischemic stroke may feature in chronic systemic inflammatory conditions (Harris et al., 2008). Changes in the diameter of the cerebral vasculature have also been observed in cases of SAH, stroke, epilepsy and migraine through propagating waves of neuronal depolarization, otherwise known as spreading depolarization (SD; Lauritzen et al., 2011). Inflammatory mediators have also been associated with waves of SD (Urbach et al., 2006), thus reinforcing the hypothesis that inflammation may have a crucial role in determining vessel contractility and tissue perfusion. However, future studies are needed to directly examine the role of chronic inflammation on CBF following stroke and brain injury and to what degree the pro-inflammatory cytokine IL-1 might play in altering vasomotor tone in chronically inflamed cerebrovasculature.

### Inflammation and hypoperfusion: a mechanical element?

Following CNS injury, a breach in endothelial integrity has multiple downstream consequences ranging from alterations in endothelial reactivity, vascular tone, pro-coagulant state and inflammatory phenotype (Taka et al., 2002; Clark et al., 2012). The cerebral endothelium is a primary target for neuroinflammatory stimuli due to its capacity to alter vascular tone through chemical and mechanical mechanisms. As discussed above the pro-inflammatory cytokine IL-1 can directly induce expression of vasoactive mediators (e.g., ET-1) which can alter vascular tone through actions on vascular smooth muscle (Moncada and Higgs, 2006; Anfossi et al., 2010). However, the cerebrovasculature can also obstruct CBF following CNS injury by mechanical means.

#### The role of leukocyte-platelet interactions on CBF

**Figure 2:**
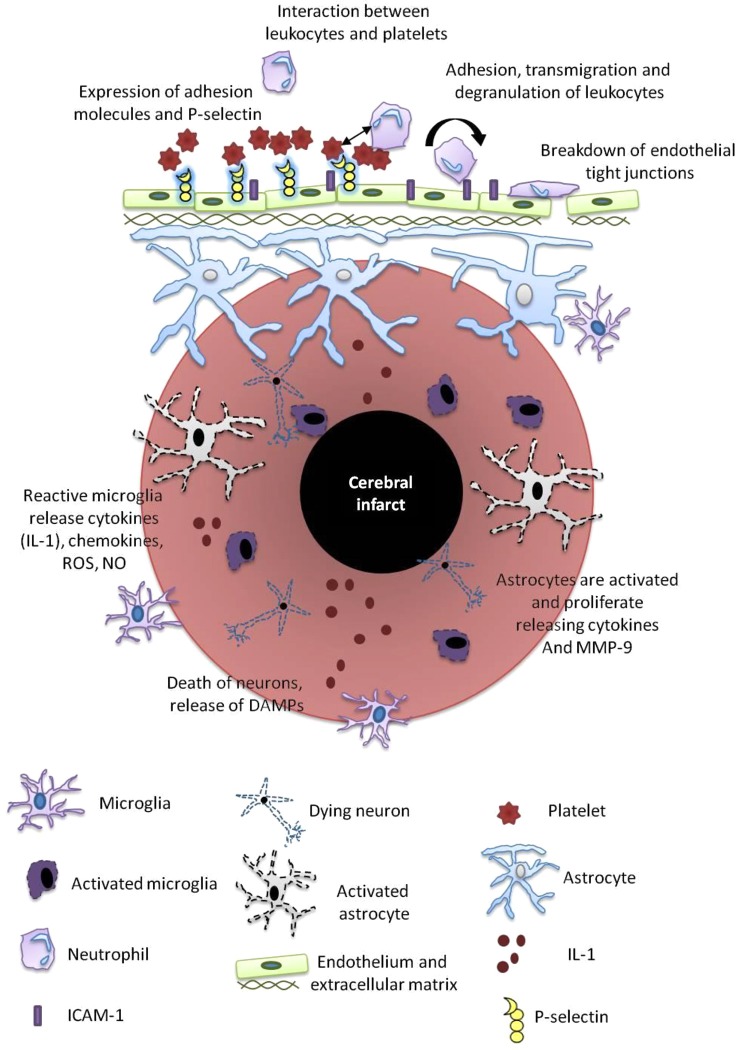
**Post-stroke inflammation**. Mechanisms of post-stroke inflammation occur via a number of pathways as outlined and include a variety of resident immune cells including microglia, astrocytes, neutrophils, platelets and the cerebral endothelium. These cells release mediators that propagate the inflammatory cascade including reactive oxygen species (ROS), nitric oxide (NO), damage-associated molecular patterns (DAMPs), metalloproteinases (MMPs), ICAM and IL-1.

Physical blockade within the cerebrovasculature can be mediated by the interaction between neutrophils and brain endothelial cells. Inflammation can induce an upregulation of adhesion molecules (P-selectin, intercellular adhesion molecule-1 (ICAM-1), vascular cell adhesion molecule-1) and chemokines (IL-8, monocyte chemoattractant protein-1, macrophage inflammatory protein-1) in endothelial cells. Within hours of ischemic injury, circulating neutrophils can either transmigrate between endothelial cells from the blood to the injured tissue or remain adherent to the luminal surface of blood vessels as seen in murine and human post-stroke tissue samples (Enzmann et al., 2013). Neutrophils can release pro-inflammatory mediators which elicit secondary injury to the salvageable ischemic penumbra (Jin et al., 2010; Figure 2). Physically, the presence of neutrophils in the microvasculature of the brain under conditions of ischemia and altered perfusion can result in an exaggerated neutrophil accumulation and obstruction in CBF, referred to as the “no re-flow” phenomenon (del Zoppo et al., 1991). In a similar fashion to neutrophils, circulating platelets can also exacerbate ischemic stroke mechanically by impeding CBF. The “no re-flow phenomenon” suggests that the post-stroke complication of hypoperfusion can be attributed to platelet and neutrophil accumulation in microvessels alongside fibrin deposition and obstructive leukocytes (del Zoppo et al., 1991). This accumulation can be reversed in mice receiving anti-glycoprotein (GP) Ib treatment (i.e., to prevent the interaction between platelets and the brain endothelium) thus showing an improvement in post-stroke ischemic CBF (Pham et al., 2011). Furthermore, in transient models of ischemia, anti-leukocyte interventions result in neuroprotection. Using laser-scanning confocal microscopy and laser-Doppler perfusion imaging, neutrophils adhering to the endothelium have been shown to contribute to perfusion deficits following the restoration of CBF (Belayev et al., 2002). Furthermore, treatment with albumin was shown to reverse the adherence and perfusion deficits within the post-capillary microcirculation during the post-ischemic reperfusion period. This mechanical obstruction of CBF by accumulation of platelets and/or neutrophils has also been seen in models of SAH in both dogs and humans (Asano and Sano, 1977; Dóczi, 1985).

In addition to their mechanical effects platelets also have detrimental chemical actions, including the ability to expel their granular contents and to synthesize immune related proteins such as IL-1 (Afshar-Kharghan and Thiagarajan, 2006). Indirectly, platelets can induce an inflammatory response in other cells (e.g., endothelium, microglia) by releasing IL-1 (Hawrylowicz et al., 1991). The important role of activated platelets has been seen in recent research showing that immediately following injury neutrophils recruited to sites of injury can extend a domain to scan for locally activated platelets. Only when productive interactions between platelets and neutrophil projections occur do neutrophils initiate intravascular migration or generate NETs to propel inflammatory responses (Sreeramkumar et al., 2014). This suggests neutrophils and platelets work co-operatively to exacerbate inflammation. *In vivo*, platelets represent a source of IL-1α and it has been proposed that activation of cerebral endothelium via platelet-dependent IL-1 is a crucial step in triggering neutrophil migration to the parenchyma (Thornton et al., 2010). Experimentally, neutralizing platelet GP surface receptors (Le Behot et al., 2014) or use of small molecule inhibitors of GpIIb/IIIa (Lapchak et al., 2002) can improve CBF and functional outcome following ischemic stroke. However, care must be taken when targeting particular GP interactions as some have more potent antithrombotic effects than others (Grüner et al., 2005). Heightened systemic inflammation can also exaggerate platelet adhesion, aggregation and the coagulation cascade (Cao et al., 2009; Granger et al., 2010) which again highlights the pivotal role that inflammatory cascades play in multiple stroke etiologies. Platelet hyperactivity and dysregulation is common to diabetes (Ferroni et al., 2004), hypercholesteremia (Haramaki et al., 2007), hypertension (Gkaliagkousi et al., 2009) and atherosclerosis (Ruggeri, 2003). Thus, platelet and leukocyte interactions are a hallmark of acute and chronic inflammatory diseases and in combination with an ischemic injury may have synergistic detrimental effects.

Another important regulator of CBF are pericytes. Pericytes are contractile cells located on capillaries and have an important role in controlling CBF. In one study exploring the role of pericytes, rat brain slices were exposed to conditions mimicking ischemia, resulting in persistent vasoconstriction and pericyte death (Hall et al., 2014). This pericyte death caused a “rigor mortis” and prolonged vasoconstriction due to adenosine triphosphate (ATP) deprivation that restricts myosin and actin separation and subsequent relaxation. Pericyte dilation and contractility is controlled by various vasoactive mediators and pericytes have the capacity to respond readily to these mediators as they are derived from the smooth muscle cell lineage (Nehls and Drenckhahn, 1993; Pieper et al., 2014). Mechanical obstruction of CBF can also occur due to compression of vessels by progressively edematous neighboring astrocytes (Ito et al., 2011).

Whilst protection of vulnerable new neurons is an important strategy in treating brain injury, stroke is, etiologically, a vascular disorder. It is therefore important to consider the implications of systemic inflammation on the cerebrovasculature and the downstream consequences on CBF.

### Repair and recovery post-stroke: the role of neurogenesis

Aside from modest advancements in neurorehabillitation therapies for stroke survivors there is an absence of effective treatment options beyond the 4.5 h time window that promote any significant recovery. Yet, the brain does command certain endogenous repair processes that are employed following CNS injury to limit cell death and promote neural repair, though this is insufficient to have any major effect in the majority of patients.

A possible driver of functional recovery is post-stroke neurogenesis. Neurogenesis is the generation of new neurons that integrate into pre-existing networks. Contrary to the historical hypothesis that neurons could only form during the developmental periods in early life and were refractory to replication, it is now well established that new neurons are continuously being created in the adult brain. This discovery was aided drastically by the advent of new techniques to track the birth and migration of new neurons (Nowakowski et al., 1989; Paez-Gonzalez et al., 2014). New neurons originate from neural progenitor cells (NPCs), defined as cells that have the capacity for self-renewal and that can produce multiple distinct cell types (e.g., neurons, astrocytes, oligodendrocytes). Adult neurogenesis is normally restricted to two neurogenic regions: the subventricular zone (SVZ; Reynolds and Weiss, 1992) and subgranular zone (SGZ; Gage et al., 1995) which anatomically house NPCs and functionally control their development. Stroke is a robust trigger of neurogenesis by stimulating NPCs of the SVZ to divide and migrate to the peri-infarct area (Arvidsson et al., 2002; Thored et al., 2006). Treatments that either increase the levels of proliferating NPCs or enhance their survival and migration to the peri-infarct brain lesion would contribute to improved functional recovery and tissue survival after stroke (Nih et al., 2012). However, although ischemic stroke promotes neurogenesis in neurogenic regions and migration of NPCs to sites of injury; most newly generated neurons fail to survive. It is proposed that inflammation associated with ischemic stroke and the pre-existing inflammatory co-morbidities or age may contribute to the high levels of apoptotic death of stroke-generated neuroblasts in preclinical models of ischemia (Seki and Arai, 1995; Kuhn et al., 1996; Kokaia et al., 2006).

### Inflammation and neurogenesis

Mechanisms through which inflammation impairs neurogenesis are poorly understood, due to the range of cells and signaling pathways that can be activated in response to an inflammatory stimulus. Adult neurogenesis is compromised in environments of the brain with mitochondrial dysfunction (Kirby et al., 2009), raised reactive oxygen species (ROS; Zhang et al., 2012), brain irradiation (Monje et al., 2003) and most interestingly, in the presence of activated microglia (Ekdahl et al., 2003; Monje and Palmer, 2003). The connection between reduced neurogenesis and an upregulation in the number and activity of microglia has been observed in response to systemic LPS and results in a 240% increase in the density of detrimental microglial cells in the dentate gyrus (DG), a structure which is essential for learning and memory formation and consolidation. Detrimental actions of microglia on neurogenesis involve production of ROS and NO (Gebicke-Haerter et al., 2001; Moreno-López et al., 2004). Ablation of microglial function using indomethacin (Hoehn et al., 2005) or minocycline (Liu et al., 2007) improves numbers of NPCs after stroke. However, complete inactivation of microglia may not always have positive effects. It has been hypothesized that microglia may be responsive to interactions with CNS-specific T-cells and thus promote NPC proliferation and neuronal survival (Ziv et al., 2006; Schwartz et al., 2009).

Angiogenesis is another important route of tissue repair post-stroke as blocking angiogenesis reduces the localization of immature neurons to peri-infarct tissue (Ohab and Carmichael, 2008). Inflammatory mediators, in particular IL-1 have been implicated in augmenting angiogenic processes. In a study by Pham et al. (2012) IL-1β stimulated oligodendrocytes to produce MMP-9 in the conditioned media. This conditioned media was placed on endothelial cell cultures resulting in a significant increase in endothelial tube formation. This process was mirrored *in vivo* whereby neutralization of IL-1β in a white mater injury model reduced oligodendrocyte MMP-9 expression and thus angiogenesis (Pham et al., 2012). This improvement in angiogenesis post-stroke following IL-1β treatment has also been seen in endothelial progenitor cell (EPC) cultures. Treatment of EPC cultures with the conditioned media from primary rat cortical astrocytes promoted EPC mediated neurovascular remodeling during the post-stroke period (Hayakawa et al., 2012).

### Anti-inflammatory strategies to improve post-stroke neurogenesis

Current drugs e.g., minocycline, that manipulate microglial function often are broad spectrum and unspecific and influence multiple inflammatory pathways essential for the repair phase of stroke recovery. Since neurogenesis may occur for up to a year following stroke, chronic administration of a drug that can inhibit repair may not be ideal. It is therefore important to consider more specific targets of inflammation rather than broad-spectrum drugs to promote neurogenesis after stroke. As indicated earlier IL-1 is implicated in learning and memory and there are numerous studies showing that stress, which involves an elevated inflammatory profile (Banasr et al., 2007) and high levels of IL-1, causes hippocampal dysfunction and a reduction in neurogenesis (Ben Menachem-Zidon et al., 2008; Mathieu et al., 2010). Preclinical data shows that IL-1 exerts anti-neurogeneic properties in chronic stress through up-regulation of NFκB, activator protein (AP)-1 and signal transducer and activator of transcription (STAT)-1 (Pugazhenthi et al., 2013). The actions of IL-1 on neurogenesis have been examined *in vitro* in primary adult hippocampal progenitor cells which possess IL-1RI (Koo and Duman, 2008) and embryonic cortical NPCs (Ajmone-Cat et al., 2010). When the adult hippocampal progenitor cells were treated with IL-1β, there was a reduction in the number of proliferating progenitor cells. Furthermore, this anti-neurogenic effect was found to be mediated by activation of the NFκB signaling pathway, and could be blocked by IL-1Ra (Koo and Duman, 2008). IL-1 also activates the endothelium to produce trophic factors such as vascular endothelial growth factor (VEGF) and insulin-like growth factor (IGF)-1, which are needed for neurogenesis (Anderson et al., 2002; Cao et al., 2004) and is also important in the reparative angiogenic process (Coxon et al., 2002; Voronov et al., 2003; Carmi et al., 2009). However, effects of IL-1 on neurogenesis following acute cerebral ischemia *in vivo* have yet to be elucidated but the potential use of IL-1Ra to improve neurogenesis is an attractive possibility. However, it must be considered that such anti-inflammatory treatments e.g., IL-1Ra, for stroke must be administered within a time frame that does not interfere with the repair process, otherwise there may be detrimental effects (Girard et al., 2014).

### Potential treatment strategies targeting the IL-1 system

**Figure 3:**
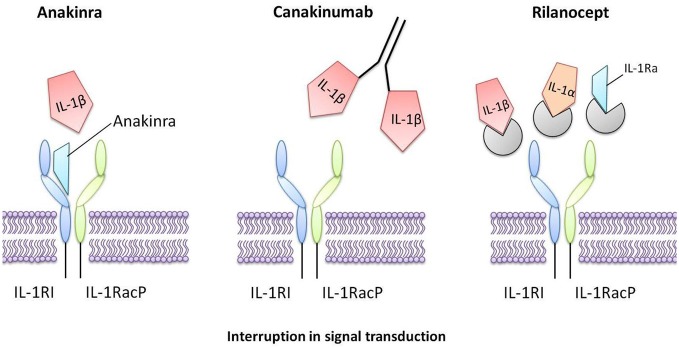
**Interleukin-1 inhibitors that are in current clinical use**. Anakinra is a recombinant form of human IL-1Ra that directly competes with IL-1 for binding to the IL-1 type I receptor, therefore blocking the biological activity of IL-1. Canakinumab is a human monoclonal antibody that selectively targets IL-1β. Rilonacept is a human dimeric fusion protein that interrupts IL-1 signaling by incorporating components of the IL-1 receptor, thus trapping and sequestering circulating IL-1.

Evidence discussed above and in many other articles highlights the IL-1 system as an attractive therapeutic target in the search for treatments for acute CNS injury. Therapeutic interventions include direct targeting of IL-1, antagonism of the IL-1 receptor, use of neutralizing antibodies and inflammasome inhibitors (Figure 3). Despite these alternatives, IL-1Ra remains the most widely researched inhibitor of IL-1 actions due to its high specificity and safety. Anakinra, the recombinant form of human IL-1Ra, has a half-life of 4–6 h and is well-tolerated in the patient population, as evidenced by significant research in the rheumatoid arthritis field in which it is a mainstream drug treatment (Mertens and Singh, 2009). Within preclinical stroke studies, the neuroprotective effects of IL-1Ra have been seen in a variety of species e.g., mice, rats and gerbils (Ohtsuki et al., 1996; McColl et al., 2007; Pradillo et al., 2012) and in differing models of ischemia e.g., focal, global, transient and global (Rothwell, 2003). IL-1Ra maintains its neuroprotective effects through a number of routes of administration e.g., i.c.v, i.v and subcutaneously (s.c) (Greenhalgh et al., 2010). Finally, and perhaps most importantly, IL-1Ra can still inhibit ischemic injury at delayed time points up to 3–4.5 h (Mulcahy et al., 2003) and in co-morbid strains. In a meta-analysis analyzing the effects of IL-1Ra in preclinical models of stroke, IL-1Ra treatment elicited an overall 38% reduction in infarct volume across 17 published studies (Banwell et al., 2009; Parry-Jones et al., 2010). In the clinical arena, results from a randomized, double-blind, placebo-controlled trial of IL-1Ra in acute stroke showed that patients receiving IL-1Ra had lower peripheral white blood cell counts, neutrophil counts, CRP and IL-6 levels. Furthermore, by 3 months, these patients showed some evidence of improved recovery compared to placebo-controlled counterparts, though it is important to realize the study was not powered for such an outcome (Emsley et al., 2005). Further larger scale trials of IL-1Ra in both SAH (ISRCTN25048895) and acute ischemic stroke (ISRCTN74236229) are ongoing. The clinical trial examining the effects of IL-1Ra on inflammatory mediators in SAH has just recruited the final patient and results are expected early in 2015. It is a multi-center, single-blind open label randomized control trial incorporating 140 patients. IL-1Ra was administered twice daily by s.c administration for 21 days in patients presenting within 72 h with aneurismal SAH. Blood samples were taken during this time period to analyze IL-6 and CRP alongside the Glasgow outcome scale. The clinical trial examining the effects of IL-1Ra in ischemic stroke started recruiting in Spring 2014 with participants receiving twice daily, s.c injection of IL-1Ra or placebo. The first injection of IL-1Ra is being given within 6 h of stroke onset with 5 more doses at 12 h intervals for 3 days.

In a clinical trial exploring the role of IL-1β in type II diabetes disease progression, in which patients received IL-1Ra (s.c) once daily for 13 weeks, an improvement in insulin production and glycemic control was observed, along with a reduction in the inflammatory biomarkers, CRP and IL-6 (Larsen et al., 2007). More promising still, in the 39 week follow up study, patients receiving IL-1Ra used 66% less insulin to return to baseline glycemic control levels (Larsen et al., 2009). In a phase II randomized control trial in patients with severe TBI (s.c) administration of IL-1Ra has been shown to be safe, penetrate the brain and to alter the neuroinflammatory response (Helmy et al., 2014).

Clinically, abrogation of IL-1β has also been explored (Yamasaki et al., 1995). Canakinumab is a human monoclonal antibody that selectively targets IL-1β and it has a half-life of 21–28 days (Chakraborty et al., 2012). The use of canakinumab in humans has already been approved for treating arthritis and tested in cryopyrin-associated periodic syndrome (CAPS; Church and McDermott, 2010; Kuemmerle-Deschner et al., 2011; Chakraborty et al., 2013). With this clinical success has come a barrage of research using this anti-IL-1β antibody with much interest in its use in neonatal onset inflammation disease (Sibley et al., 2014), type II diabetes (Howard et al., 2014) and stroke (Ridker et al., 2011). Direct targeting of IL-1 has also been achieved using rilonacept, a human dimeric fusion protein that interferes with IL-1 signaling due to the presence of extracellular components of IL-1RI and IL-1RaP which bind with high affinity to circulating IL-1. This “IL-1 trap” has a half-life of 67 h and has been shown to be safe and effective in CAPS (Goldbach-Mansky et al., 2008; Hoffman et al., 2008). It has been shown that directly targeting IL-1 is a clinically approved strategy for treating auto-immune and autoinflammatory diseases. However further pre-clinical and clinical research is needed if these inhibitors are to be used as therapeutic agents in treating stroke or acute brain injury. Another important consideration when targeting IL-1 is the relative contribution of IL-1α vs. IL-1β. IL-1Ra and rilonacept block the actions of both α and β however canakinumab only targets IL-1β. Historically, IL-1β was considered the primary ligand mediating an exaggerated response to ischemic injury however recent research indicates IL-1α also plays a crucial role in post-stroke pathogenesis and that it may proceed IL-1β expression (Luheshi et al., 2011). In conclusion, it is essential to consider the relative contribution of IL-1α and β to the disease in question, and to identify the time frame in which the anti-inflammatory strategy may be of most benefit.

One of the disadvantages of using IL-1Ra is that BBB penetration is poor due to the large size (kDa) of the macromolar protein. An alternative could be the use of a novel, synthetic peptide called Llantide. This protein is derived from the N-terminal domain of IL-1Ra and therefore mediates its protective effects by binding to IL-1RI and therefore inhibiting NFκB activation and secretion of TNFα from macrophages. The use of this novel peptide in response to an inflammatory challenge e.g., LPS, reduces symptoms of sickness behavior and reduced social depression commonly associated with systemic LPS administration alongside improving plasma levels of IL-10 (Klementiev et al., 2014). Small molecule inhibitors of caspase-1 are protective in experimental models of acute CNS injury (Ross et al., 2007; Suzuki et al., 2009), while neutralization of components of the NLRP1 inflammasome is beneficial in rodent models of ischemic stroke (Fann et al., 2013). Furthermore, ablation of components of the NLRP3 inflammasome are associated with reduced leukocyte infiltration, reduced edema and improvements in neurological function following ICH in mice (Ma et al., 2014). However inhibition of caspase 1 or the inflammasome has yet to be evaluated clinically in stroke.

### Concluding remarks

A wealth of evidence now exists to show that inflammation is a critical component in cerebral ischemia, by increasing risk and contributing to worse outcome. Conversely, late stage inflammatory processes post-stroke may contribute to brain repair. IL-1 is the first described inflammatory cytokine and has numerous actions that contribute to both injury and repair. Block of IL-1 actions has been demonstrated to be effective in a wide range of clinical conditions, and there is strong experimental evidence to support its role as a key mediator of acute neuronal injury. Ongoing clinical trials in stroke and SAH using IL-1Ra to block the effects of IL-1 will provide further evidence on the potential of IL-1 as a target. Ultimately though this will only be confirmed following successful large Phase III clinical trials of IL-1Ra or alternative inhibitors of IL-1 actions.

## Conflict of interest statement

The authors declare that the research was conducted in the absence of any commercial or financial relationships that could be construed as a potential conflict of interest.
